# The universal role of adaptive transcription in health and disease

**DOI:** 10.1111/febs.17324

**Published:** 2024-11-28

**Authors:** Thomas Lissek

**Affiliations:** ^1^ Interdisciplinary Center for Neurosciences Heidelberg University Germany

**Keywords:** adaptation, disease, health, maladaptation, plasticity, transcription

## Abstract

In animals, adaptive transcription is a crucial mechanism to connect environmental stimulation to changes in gene expression and subsequent organism remodeling. Adaptive transcriptional programs involving molecules such as CREB, SRF, MEF2, FOS, and EGR1 are central to a wide variety of organism functions, including learning and memory, immune system plasticity, and muscle hypertrophy, and their activation increases cellular resilience and prevents various diseases. Yet, they also form the basis for many maladaptive processes and are involved in the progression of addiction, depression, cancer, cardiovascular disorders, autoimmune conditions, and metabolic dysfunction among others and are thus prime examples for mediating the adaptation–maladaptation dilemma. They are implicated in the therapeutic effects of major treatment modalities such as antidepressants and can have negative effects on treatment, for example, contributing to therapy resistance in cancer. This review examines the universal role of adaptive transcription as a mechanism for the induction of adaptive cell state transitions in health and disease and explores how many medical disorders can be conceptualized as caused by errors in cellular adaptation goals. It also considers the underlying principles in the basic structure of adaptive gene programs such as their division into a core and a directional program. Finally, it analyses how one might best reprogram cells via targeting of adaptive transcription in combination with complex stimulation patterns to leverage endogenous cellular reprogramming dynamics and achieve optimal health of the whole organism.

## Graphical abstract

Adaptive transcription via molecules such as CREB, MEF2, SRF, AP1, Nr4a1, and Egr1 constitutes a universal mechanism for directed cell state transitions in health and disease. These molecules are involved in physiological adaptation processes, pathological maladaptations, curative adaptations, and development of resistance to treatment. Decoding the logic behind adaptive transcription programs will uncover fundamental organizational principles of biological systems and facilitate the development of novel therapeutic concepts for a variety of diseases.

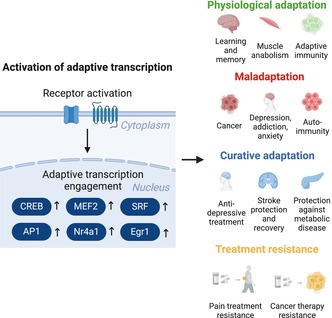

**Abbreviations**
AML: acute myeloid leukemia
AP1: activator protein 1
ATF: activating transcription factor
BDNF: brain‐derived neurotrophic factor
cAMP: cyclic adenosine monophosphate
CaN: calcineurin
CBP: CREB‐binding protein
CREB: cAMP response element‐binding protein
CRTC: CREB‐regulated transcription coactivator
ECT: electroconvulsive therapy
EGR: early growth response
Fndc5: fibronectin type III domain‐containing protein 5
FOS: Fos proto‐oncogene
HiK: high potassium chloride depolarization
ICER: inducible cAMP early repressor
IEG: immediate early gene
IL: interleukin
MAPK: mitogen‐activated protein kinase
MCT1: monocarboxylate transporter 1
MEF2: myocyte enhancer factor 2
MSN: medium spiny neuron
NFAT: nuclear factor of activated T cells
NF‐kB: nuclear factor kappa B
NMDA: *N*‐methyl‐d‐aspartate
NR4A: nuclear receptor subfamily 4A
PDGF: platelet‐derived growth factor
Pgc1a: peroxisome proliferator‐activated receptor gamma coactivator 1‐alpha
PKA: protein kinase A
SRF: serum response factor
TNF: tumor necrosis factor
UTP: uridine triphosphate

## Introduction

The ability to adapt to complex environments is a hallmark of living systems. Animals in particular have to tune their components at various levels (molecules, cells, tissues, organs, organ systems, whole body) to defined parameters in the environment. At the cellular level, this constitutes a reprogramming mechanism which connects environmental stimulation to changes in cellular physiology (e.g., modifying excitability of a neuron through differential ion channel subunit expression while encoding a memory). In mammals, this cellular reprogramming is regulated by a set of transcription factors that include cAMP response element‐binding protein (CREB) and its coactivators (most notably CREB‐binding protein (CBP) and CREB‐regulated transcription coactivator (CRTC)), serum response factor (SRF), myocyte enhancer factor 2 (MEF2), and immediate early genes (IEGs) such as family members of activator protein 1 (AP1) (e.g., Fos and Jun), early growth response (EGR) (e.g., Egr1), nuclear receptor subfamily 4A (NR4A) (e.g., Nr4a1 and Nr4a3), and activating transcription factor (ATF) (e.g., Atf3) among others. These genes are ubiquitously expressed and their protein products active throughout the mammalian body. They are involved in a wide variety of adaptive processes such as learning and memory, immune defense, skeletal muscle hypertrophy, and metabolic adaptation to nutritional challenge.

Transcriptional programs which are induced by acute stimulation and control remodeling of cells to adapt to changing environmental conditions are termed *adaptive transcription* here. Adaptive transcription via the molecules mentioned above thus distinguishes itself from constitutive transcription (e.g., housekeeping genes) or less dynamical transcriptional changes (e.g., inducible but afterward stable expression of cell‐type defining genes during development). Adaptive transcription is highly plastic (e.g., different inputs lead to different gene induction patterns) and dynamic (e.g., it can be induced and shut‐off on the minute timescale). The primary distinguishing marks of adaptive transcription are that its components are acutely induced by cellular stimulation (i.e., in the absence of relevant cellular stimulation, adaptive transcription programs are not expressed or only at low steady‐state levels) and that it is required for certain long‐term cellular adaptation mechanisms.

Previous work has identified adaptive gene programs as central to inducible health improvements through physical and cognitive stimulation and has proposed that they represent universal targets to partially rejuvenate adult somatic cells [1]. However, as we will see below, in spite of their important role in maintaining and improving health, these gene programs are also crucially involved in the pathogenesis of many of humanity's gravest diseases including several psychiatric conditions, cancer, cardiovascular disorders, metabolic dysfunction, and autoimmunity. The involvement of adaptive transcriptional programs in processes both beneficial and detrimental to human health represents an interesting example of the adaptation–maladaptation dilemma [2, 3]. How can these molecular programs simultaneously protect against disease as well as drive disease progression? Below, I will outline a potential answer that frames maladaptive disorders as caused by errors in the adaptive logic of organisms and it will be explored how one could correct these logic errors through complexity‐preserving reprogramming.

**Fig. 1:**
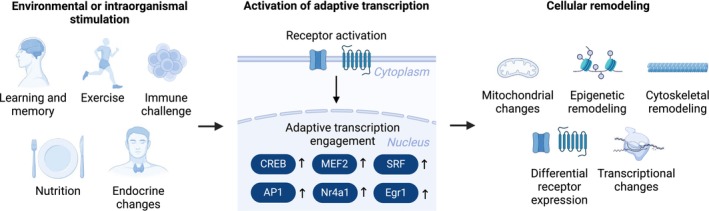
Adaptive transcription is activated by cellular stimulation and mediates cell remodeling. Environmental or intraorganismal stimulation is translated to stimulation of receptors via signaling molecules or opening of voltage‐sensitive ion channels through membrane depolarization which then initiates signaling cascades to the nucleus. In the nucleus, adaptive transcription factors such as cAMP response element‐binding protein (CREB), serum response factor (SRF), myocyte enhancer factor 2 (MEF2), activator protein 1 (AP1), and early growth response 1 (Egr1) are activated. Adaptive transcriptional programs mediate diverse cellular remodeling processes such as opening of chromatin, changes in epigenetic marks, tuning mitochondrial metabolism, remodeling cytoskeletal structure, regulating channel and receptor expression and changing transcriptional programs over the long‐term. This remodeling at the cellular level translates to changes in organism function and behavior.

We will focus our discussion on representative molecules, including CREB, SRF, and MEF2 at the level of activity‐regulated transcription factors and AP1 members (e.g., Fos and Jun) and Egr1 at the level of IEGs, and explore closely related genes and proteins where indicated (e.g., CRTC, Npas4, Nr4a3). There are additional molecules in the class of adaptive transcription components including NFAT [4], NF‐kB [5], and Pgc1a [6] among others that cannot be treated here in detail due to space limitations. Rather the molecules above serve as examples to extract underlying principles that could be extended to more constituents of adaptive transcription programs in future research. Figure 1 gives a brief overview of the mechanism of adaptive transcription.

We will also focus our discussion on a select group of processes in health and disease and note that adaptive transcription has roles in many more bodily processes than are mentioned here. For each organ system or disease entity being discussed, only selected references can be given as comprehensively reviewing each entity would be beyond the scope of the present work.

## Adaptive transcription in health

**Fig. 2:**
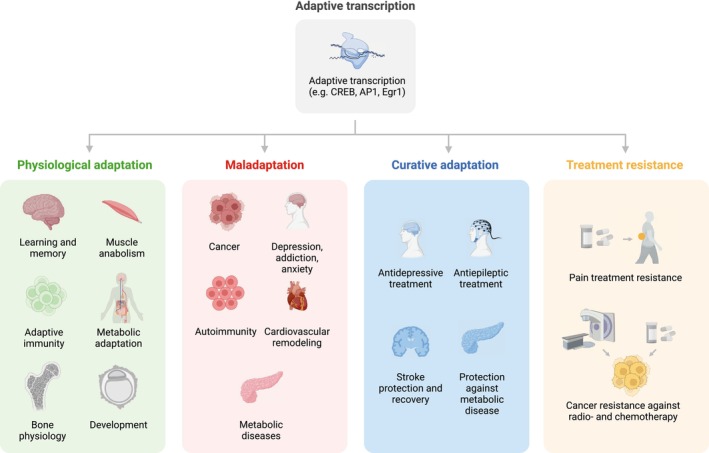
Adaptive transcription mediates diverse physiological, pathological, and treatment‐relevant adaptation processes. Adaptive transcription mediates a diverse set of physiological and pathological plasticity processes. In health, these include learning and memory, immune defense, muscle hypertrophy, metabolic plasticity, skin adaptation and wound healing, as well as bone physiology. Adaptive transcriptional programs also mediate maladaptive processes such as addiction, depression and anxiety, cancer, cardiovascular diseases, autoimmune diseases, and metabolic disorders. In treatments, adaptive transcription can both mediate curative effects (e.g., effects of antidepressants) and treatment resistance (e.g., resistance against radiotherapy).

Adaptive transcription programs mediate a wide variety of cellular plasticity processes throughout the whole body (Fig. 2).

### Learning, memory, and brain physiology

In the brain, the encoding of memories within neural circuits requires functional and structural changes to their cellular components and adaptive transcription is central to this plasticity. CREB is phosphorylated in the rodent brain *in vivo* through exposure to learning tasks [7] and *in vitro* in neurons in response to stimuli that induce synaptic plasticity [8, 9]. Studies in which CREB expression or activity have been reduced have implicated it in various physiological forms of learning and memory [10, 11, 12, 13]. Conversely, expression of a constitutively active form of CREB or overexpression of CREB enhances memory in several paradigms [14, 15, 16, 17, 18, 19]. At the cellular level, CREB regulates neuronal excitability [14, 20, 21], dendritic growth [22], and synaptic function [21, 23] among other parameters. SRF is induced by neuronal depolarization or neurotransmitter application [24] and is involved in memory formation [25] and synaptic plasticity [25, 26]. MEF2 is implicated in learning and memory [27] and synapse formation [28]. Fos is induced in the rodent brain *in vivo* through learning tasks [29, 30, 31] and has been shown to be causally involved in memory formation [32, 33, 34] as well as experience‐dependent neural circuit remodeling [35, 36]. Similarly, Egr1 is induced by learning tasks [30] and involved in memory formation [37, 38] as well as synaptic plasticity [38]. Npas4 is induced by memory tasks and involved in memory formation [39] and shapes neural circuit function [40, 41, 42]. In the brain, adaptive transcription hence mediates the transition from environmental stimulus to the recoding of neural network function and structure that represents memory formation. It enhances overall cellular plasticity but also directs cell function changes into concrete directions (e.g., see Npas4's function in different cell types [40]).

### Skeletal muscle plasticity and regeneration

A critical function in mammals is adaptation of motor function to high demand, in part through skeletal muscle hypertrophy and regeneration after injury. Human studies have shown that exercise activates or increases adaptive transcriptional components in skeletal muscle, including CREB [43], MEF2 [44], and several IEGs [45, 46]. In rodents, CREB is involved in muscle regeneration [47], CRTC2 mediates muscle anabolism [48], and SRF is required for adaptive muscle hypertrophy [49, 50]. Expression of constitutively active MEF2 induces muscle hypertrophy [51], and MEF2 is required for skeletal muscle regeneration after injury [52]. Fos is involved in muscle regeneration after injury [53] and Junb regulates muscle mass in rodents with its overexpression inducing hypertrophy [54]. Nr4a3 is induced in human muscle through exercise [45] and regulates the molecular response of muscle cells to exercise‐mimicking stimuli *in vitro* [55]. Nr4a3 overexpression increases type 2 muscle fibers and fatigue resistance in mice *in vivo* [56].

### Immune function

Immune cells have to constantly reprogram themselves in the course of immune defense and adapt to changing antigen exposure, for which they employ adaptive transcription programs. In mice, CREB is needed for thymocyte proliferation and IL‐2 induction [57], physiological Th cell function [58], and B‐cell function [59]. MEF2 family members are involved in various immune cell processes such as T‐cell cytokine regulation [60], T‐cell apoptosis [61], Treg cell activation [62], B‐cell development [63], B‐cell proliferation [64], and macrophage polarization [65]. SRF is required for thymocyte‐positive selection and the development of T reg cells [66], as well as various other functions during lymphocyte development [67]. AP1 opens chromatin during T‐cell activation [68] and Fos has been proposed to carry a short‐term memory signal in T cells [69]. Egr1 is involved in B‐cell function [70, 71] and in Th2 cell IL‐4 transcription [72].

### Cardiovascular physiology and adaptation

The cardiovascular system has to continuously adapt to changes in the environment (e.g., heightened requirements for exercise) and organism (e.g., stress hormone levels). In the heart, CREB maintains ventricular function [73], cardiomyocyte electrophysiology [74], and mitochondrial function [75]. In humans, the CREB1 gene sequence is a genetic predictor of the heart rate response to regular exercise [76], and in mice, CREB has been linked to adaptive hypertrophy after exercise [77]. In vascular smooth muscle cells, CREB regulates hypertrophy in response to angiotensin II [78], as well as proliferation under basal conditions [79] and in response to PDGF [79], angiotensin II [80], and thrombin [81]. It also controls vascular smooth muscle cell migration in response to TNF‐alpha [82] and UTP [83]. In cardiomyocytes, SRF regulates genes that are known to be involved in hypertrophy [84], its overexpression leads to cardiac hypertrophy [85], it regulates the function of the contractile apparatus [86], and its deletion in adult cardiomyocytes *in vivo* leads to dilated cardiomyopathy [87]. MEF2 protects cardiomyocytes from cell death [88, 89] and regulates mitochondrial and cytoskeletal physiology [90]. Nr4a1 protects against various forms of adverse cardiac remodeling [91, 92, 93].

### Pancreas, liver, and adipose tissue physiology

Pancreas and liver cells have to adapt their function to the nutritional status of the organism, especially with regard to glucose regulation. In pancreatic beta cells, CREB is induced by glucose stimulation and promotes cell survival [94] and proliferation [95] and mediates cAMP‐induced transcriptional changes [96]. In the liver, CREB is regulated by glucagon and insulin *in vivo* and controls gluconeogenesis [97]. CRTC regulates hepatic gluconeogenesis [98] and adrenergic signaling in adipose tissue [99]. SRF controls in beta cells the transcription of insulin [100]. Fos is upregulated in beta cells in response to stimulation with glucose and cAMP [101] and is a critical regulator of beta cell insulin secretion and cell proliferation [102]. Similarly, Egr1 is induced in beta cells by metabolic stimulation [101, 103] and regulates insulin gene transcription [104]. Nr4a1 and Nr4a3 were shown to regulate beta cell mitochondrial physiology and insulin secretion [105], as well as proliferation [106]. Npas4 protects beta cells against cytotoxic stimuli [107], is involved in metabolic maintenance *in vivo*, and exerts protection against diabetes [108].

### Skin physiology and wound healing

The skin has to undergo complex adaptations in response to external and internal stressors, including melanogenesis after UV exposure or wound healing after injury. Melanogenesis in mice is regulated by the CREB‐associated protein CRTC [109, 110]. SRF in keratinocytes is involved in correct skin development [111]. AP1 complex proteins are induced in wound healing in humans *in vivo* [112] and in mice regulate target genes that are known to be involved in wound healing [113]. Junb has been shown to be important in physiological wound healing [114] and for the physiology of the epidermo‐pilosebaceous unit in the skin [115]. cJun is important in regulating the epidermal leading edge [116]. AP1 proteins are induced in human keratinocyte differentiation [117]. Egr1 is required for skin fibroblast migration and wound healing [118].

### Bone and cartilage physiology

Bone tissue is continuously remodeled in mammals in line with hormone status and environmental demands. CREB in chondrocytes is critical for hypertrophy and bone formation [119]. MEF2 is involved in cartilage function in mice and regulates chondrocyte hypertrophy and bone length [120]. Fos is a critical regulator of osteoclast development [121] and bone remodeling [121, 122]. In mice, Fosl1 knockout produces an osteopenia phenotype in part due to a reduction in bone matrix formation [123]. Fosl2 in osteoblasts mediates differentiation and Fosl2 overexpression produces an osteosclerotic phenotype [124]. AP1 has been implicated in chondrocyte hypertrophy [125]. Atf3 regulates osteoclast precursor proliferation and bone remodeling [126].

### Development

Most of the examples above were given for functions in adult tissues and in the following section a few examples of the role of adaptive transcription in development will be given. CREB is involved in general embryonic and nervous system development [127, 128], MEF2 in the development of skeletal muscle [129, 130], the cardiovascular system [131, 132, 133], and bone tissue [120]. SRF is involved in development of the neural crest [134], neuronal tissue [135], and the heart [136], while Fos is involved in brain development [137] and bone development [122].

## Adaptive transcription in disease

In general, the potential type of involvement of adaptive transcription in disease is twofold. First, in some disorders, adaptive transcription could simply be downregulated or inhibited leading to a lack of its adaptation and pro‐survival functions and to a degradation of physiological organism function. As the treatment approaches are fairly straightforward, at least in theory, we will not discuss this category in detail here. Second, adaptive transcription could mediate maladaptation, that is, it functions ‘correctly’ or is overactivated but reprograms cells into dysfunctional states. This group of diseases is more interesting as it uses the same adaptive mechanisms that are used during physiological remodeling, just seemingly in a wrong direction, leading to overall organism dysfunction. We will focus here on one or a few representative diseases for each organ system. For instance, in the nervous system, we discuss addiction, depression, and anxiety as models for maladaptation while noting that adaptive transcription is implicated in several other psychiatric disease entities.

### Addiction

Addiction can be seen as a pathological form of learning and, not surprisingly, adaptive transcription plays a central role in this form of maladaptation. CREB has a complex role in addiction, with its activity in the nucleus accumbens being inversely correlated to cocaine conditioned place preference [138] but its overexpression being positively correlated with cocaine self‐administration [139]. In transgenic mouse models, CREB activity antagonizes the rewarding effects of cocaine [140] and a decrease in CREB activity attenuates morphine withdrawal symptoms [141]. SRF regulates cocaine‐induced spine remodeling in medium spiny neurons (MSNs) and the locomotor response to cocaine [142] as well as deltaFosb expression after cocaine treatment [143]. MEF2 regulates cocaine‐induced spine density changes in MSNs and the locomotor response to cocaine [144]. Fos regulates cocaine‐induced dendritic remodeling of MSNs and cocaine‐related long‐term behavioral changes [145]. DeltaFosb, an alternative splice variant of Fosb, regulates cocaine‐induced locomotor behavior [146] and reward [140]. Npas4 controls several parameters of medium spiny neuron physiology and cocaine‐induced locomotor responses [147], as well as conditioned place preference [148].

### Depression and anxiety

Depression and anxiety are oftentimes the result of maladaptation. In animals for instance, learned helplessness is a model system for depressive states. Overexpression of CREB in the nucleus accumbens of mice enhances depression‐like behavior whereas expression of mCREB exerts antidepressant‐like properties [149]. Similar observations have been made in rats [150]. CREB deletion has been shown to confer resilience to stress [151], and CREB overexpression in the amygdala can exert depressive and anxiety‐like effects [152]. SRF deletion in glutamatergic neurons induces a reduction in anxiety‐like behavior in mice [153]. Npas4 mediates anxiety‐like behavior at least in part through regulating hippocampal interneuron electrophysiology [154].

### Cancer

A primary hallmark of cancer cells is their adaptability which allows them to evade immune attacks and treatment efforts (e.g., through drug resistance), as well as survive in changing conditions within the body in the course of metastasis. In human acute myeloid leukemia patients, CREB expression is associated with worse outcomes [155] and CREB promotes myeloid cell proliferation and survival in AML cells [156]. CREB promotes survival and cell growth in lung cancer cells [157, 158] and in esophageal squamous cell carcinoma cells [159]. CREB expression is associated with worse clinical outcomes in epithelial ovarian cancer [160] and hepatocellular carcinoma [161] and drives hepatocellular carcinoma progression [162]. In renal cell carcinoma, CREB promotes cell proliferation [163] and metastasis [164]. CREB has been implicated in pancreatic cancer progression and clinical outcome [165], as well as carcinogenesis in colorectal cancer [166, 167], bladder cancer cells [168], mesothelioma cells [169], melanoma cells [170, 171], and glioma cells [172]. MEF2 activity is implicated in T‐cell acute lymphoblastic leukemia [173], AML invasiveness [174], pancreatic cancer cell proliferation [175], and promotion of tumorigenicity in rhabdomyosarcoma cells [176]. Fos is able to transform cells *in vitro* [177, 178] and drive tumorigenesis *in vivo* [179, 180]. It drives proliferation, migration, and invasion of osteosarcoma cells [181], mediates radioresistance in glioma [182], is involved in the development of skin cancer [183], and enhances mammary carcinoma cell invasiveness [184]. AP1 promotes tumorigenesis *in vivo* [185], proliferation and migration in breast cancer cells and breast cancer growth [186]. Egr1 is involved in prostate cancer cell migration [187].

### Pathological cardiovascular adaptation

The cardiovascular system can develop several maladaptations including pathological cardiac hypertrophy and changes in the tissue structure of blood vessels (e.g., atherosclerosis). The endogenous CREB inhibitor Inducible cAMP early repressor (ICER) is a negative regulator of isoproterenol‐ and phenylephrine‐induced cardiac hypertrophy [188]. In mice, MEF2 is a critical mediator of cardiac hypertrophy in response to pressure overload and chronic adrenergic stimulation [189]. SRF in cardiomyocytes is involved in the induction of genes known to regulate cardiac hypertrophy [84] and SRF overexpression in the heart results in pathological cardiac remodeling including cardiac hypertrophy [85]. SRF has also been implicated in hypertension‐associated changes in vascular smooth muscle cell stiffness [190]. Fos has been implicated in the formation of atherosclerosis [191]. Inhibition of cJun activity in cardiomyocytes inhibits hypertrophy induced by endothelin 1 and phenylephrine [192] and cJun is required *in vivo* for pressure overload‐induced hypertrophy while protecting against fibrosis and myocyte apoptosis [193]. AP1 is involved in alpha‐adrenergic hypertrophy of cardiomyocytes [194] and inhibition of JunD, a negative regulator of AP1 activity, leads to heightened pressure overload‐dependent apoptosis, angiogenesis, and hypertrophy in the heart [195]. Egr1 is involved in cardiac hypertrophy in response to adrenergic stimulation [196] and has been implicated in atherosclerosis [197]. Nr4a1 is involved in cardiac remodeling after pressure overload [91] and in response to angiotensin II [198].

### Autoimmune disorders

In autoimmune disorders, immune cells start attacking the body's own tissues which is oftentimes mediated by the acquisition of faulty cellular programming. CREB has been found to be central to certain autoimmune processes by positively regulating Th17 and negatively regulating Treg cell differentiation [199] and it is involved in mediating T‐cell‐dependent colitis in an animal model [199]. CRTC2 similarly promotes Th17 cell differentiation and its downregulation protects against autoimmune encephalitis in a mouse model [200]. CBP inhibition decreases IL‐17A secretion in human cells derived from patients with ankylosing spondylitis or psoriatic arthritis [201]. MEF2 is involved in macrophage polarization and Th1 responses and its downregulation protects against dextran sulfate sodium‐induced colitis *in vivo* [65]. Fos is involved in arthritic joint destruction [202, 203], and Fosl2 drives autoimmunity by influencing Treg development [204].

### Diabetes and obesity

In type 2 diabetes, one of the hallmarks is acquired insulin resistance. In obesity, adipocyte CREB drives insulin resistance and transgenic mice expressing dominant‐negative CREB show increased insulin sensitivity [205]. Constitutively active CRTC2 increases hepatic insulin resistance and gluconeogenesis [206]. SRF is involved in diabetic nephropathy through the induction of an endothelial–mesenchymal transition of glomerular endothelial cells [207]. Egr1 mediates retinal vascular dysfunction in diabetes mellitus [208] and proliferation of glomerular mesangial cells in response to high glucose [209]. Egr1 knockout mice show protection from diet‐induced obesity, fatty liver, hyperinsulinemia, and hyperlipidemia [210] suggesting that Egr1 is a causal factor in these symptoms.

## Curative adaptation and treatment resistance

So far, we have seen that adaptive transcription mediates various aspects of healthy organism function and that it is involved in the pathogenesis of several diseases. It also has important roles in various treatment approaches and has been shown to bring diseased organism function back to healthy levels. However, adaptive transcription components were also found to counteract various treatment efforts by mediating tolerance and resistance to chemotherapy and radiation therapy. In this section, we will explore the role of adaptive transcription in therapeutic approaches.

### Adaptive transcription exerts protective and curative effects

Adaptive transcription components can exert powerful curative and protective effects when overexpressed or activated by themselves and they mediate the effects of several treatment modalities. CREB overexpression in the dentate gyrus of rats induces an antidepressant effect [211] and its overexpression in CA1 counteracts age‐related long‐term memory deficits [212]. CREB overexpression in the nucleus accumbens reduces anxiety in socially isolated animals [213], and nucleus accumbens CREB mediates the effects of antidepressant treatment [214]. In mice, AP1 activity mediates the antidepressant effect of fluoxetine [215] and BDNF has been shown to exert antidepressant effects [216]. CREB also enhances neural circuit and behavioral recovery after stroke [217] and several IEGs protect against stroke‐induced neurodegeneration [218], including Npas4 [218, 219]. Npas4 protects against chemically induced epilepsy [220], and MEF2 is induced in the brain by environmental enrichment and mediates resilience to neurodegeneration [221]. Tumor‐suppressive effects are mediated by Egr1 [222, 223] and Atf3 [224, 225]. CRTC1 exerts a protective effect against cardiac hypertrophy [226], and Jun has cardioprotective effects in a pressure overload mouse model of cardiomyopathy [193]. Protective effects against autoimmunity are exerted by p300 [227], CBP and p300 [228], Egr2 [229], Nr4a1 [230], and Nr4a3 [231]. Suppression of hepatic gluconeogenesis by metformin and insulin is mediated through CBP [232]. In pancreatic beta cells, protective effects are exerted by CREB [94], Atf3 [233], and Npas4 [107]. Beta cell Npas4 protects mice against the development of type 2 diabetes [108].

### Adaptive transcription counteracts treatment efforts

Adaptive gene programs are involved in homeostatic compensation of external stressors and can hence counteract various treatment efforts, which cells oftentimes treat as disturbances. CREB has been shown to mediate morphine tolerance in the dorsal horn of rats [234]. Nucleus accumbens deltaFosb has been shown in mice to reduce the sensitivity to analgesic effects of morphine and to accelerate analgesic tolerance development [235]. CREB, Fos, and Nr4a1 promote resistance to MAPK inhibition in melanoma [236], and CREB is activated in leukemia cells upon radiation and mediates subsequent survival [237]. Similarly, CREB mediates radiation‐induced neuroendocrine differentiation in prostate cancer cells [238] and reducing CREB expression makes prostate cancer cells more susceptible to radiation‐induced cell death [239]. SRF is involved in mediating a drug resistance phenotype in basal cell carcinoma [240] and MEF2 promotes chemotherapy resistance in AML [241] and hepatic cancer cells [242].

## The logic of adaptive transcription in health and disease

With the evidence presented above, we can now extract some underlying principles and formulate a general logic for adaptive gene programs.

In broad terms, adaptive transcription facilitates and directs cell state transitions. Adaptive transcription components can be considered molecular change mediators, meaning they impart on cells the ability to reprogram themselves and change various aspects of their physiology, especially in the long‐term. Their activation leads to widespread and coordinated cellular modifications, such as alterations in chromatin accessibility [243], epigenetic states [244, 245, 246, 247], mitochondrial function [248], cell–cell communication [249], cytoskeletal remodeling [250], ion channel signaling [147], and extracellular matrix remodeling [251].

### Activation of adaptive transcription

Adaptive gene programs are regulated by diverse cellular inputs in differential ways and provide an interesting, tangible experimental base to study scale‐bridging problems in biology (i.e., how do complex environmental or intraorganismal inputs translate over cellular signals to molecular changes and back). One central problem is how adaptive transcription is engaged, that is, what stimulus parameters have to be fulfilled for cells to express these gene programs.

One such parameter is stimulus type. Npas4 in neurons is induced only by certain stimuli (e.g., depolarization) and not others (e.g., D1 dopaminergic signaling) even through both stimulus types induce Egr1 [147]. This differential nature of Npas4 induction is already present in the upstream signaling cascades as Npas4 is independent of MAPK and PKA pathways (on both of which Egr1 induction depends) but instead depends on calcineurin (CaN) (on which Egr1 does not depend) [147]. This differential induction is mirrored at the behavioral level as cocaine application induces Fos in both the ventral and dorsal striatum but Npas4 only in the ventral striatum [147]. Similar results have been reported for other stimulus types and genes (e.g., differential Fos enhancer activation by different neurotransmitters and neurotrophins [252], and differential induction of IEGs according to anatomical region and cell type [253]). Fos induction in hippocampal neurons during a spatial learning challenge is crucial for establishing a place code and coherent tuning properties across a neuronal population [35]. The CREB coactivator CRTC2 can integrate Ca^2+^‐ and cAMP‐signaling to function as a coincidence detector for different cellular stimuli such as glucose and hormones and drive transcription accordingly [254], thus potentially tuning transcription programs in islet cells to whole organism energy metabolism. In line with this reasoning, CRTC2 knockout mice display reduced insulin transcription in the pancreas and reduced blood insulin levels [255].

Another factor is signaling dynamics with duration and amplitude of the stimulus determining the activation of adaptive transcription factors [256]. For instance, in neurons an electrical stimulus with a duration of 180s can drive sustained CREB activation and induce Fos expression, whereas an 18s stimulus only leads to transient CREB activation with no subsequent Fos expression [257]. Mild depolarization via 20 mm potassium chloride (HiK) induces MAPK phosphorylation only weakly and leads to transient CREB phosphorylation whereas depolarization with 90 mm HiK leads to robust phosphorylation of MAPK and sustained phosphorylation of CREB [258].

The activation of adaptive gene programs is thus dependent on stimulus type (e.g., the combination of neurotransmitters that is received), stimulus duration (e.g., how long the neurotransmitter binds to the receptor), and stimulus intensity (e.g., how much neurotransmitter is present). This combinatorial nature suggests the possibility of mapping cell signaling inputs to transcriptional outputs and perhaps even the inference of past stimulation patterns from transcriptome data, as has been previously suggested for synthetic interfaces between neuronal membranes and nucleic acids [259].

### Transcriptional induction of maladaptation

In health, adaptive transcriptional reprogramming enables various physiological adaptation functions such as memory formation and immune defense and increases protection against degenerative disorders. However, these gene programs can become misdirected in pathological processes and when this happens, they represent powerful mechanisms to drive organism dysfunction. The same processes that allow neurons to remodel themselves and increase their resilience in times of heightened metabolic demand (e.g., CREB activation) also increase the adaptability and survival of cancer cells. It thus seems that in many pathological conditions the goal states of cells are reset such that they reprogram themselves into suboptimal states that decrease overall organism function through the use of otherwise beneficial gene programs. Perhaps then, certain diseases might not, or not only, be the result of damage or degradation but instead be caused by ‘logic errors’ that lead physiological adaptive processes into states that cause pathological organism remodeling. It has recently between proposed that this adaptation–maladaptation dilemma is a central part of the aging process [2, 3].

A central problem in this regard then is how cells are directed toward certain states, that is, what drives them toward changing their physiology in defined ways and how are these goals implemented or represented at the molecular level. For instance, if we presume that cardiovascular diseases such as chronically elevated blood pressure are, at least in part, adaptive reactions to inputs from the environment (e.g., nutrition and mental stressors), we can leverage this knowledge to design prevention, screening and treatment approaches, and reprogram the human organism out of a dysfunctional state into a functional one by acting on it at various levels. Thus, one important problem is how adaptive transcription determines the goal state of remodeling and how it implements the measures to achieve those goals. Here, the differential gene induction mechanisms that were discussed in the previous paragraphs could give some insight. As different cellular stimulation patterns elicit distinct transcriptional responses (e.g., also see different cellular responses to the same molecules activated with different time courses [256]) and subsequent remodeling, mapping these input–output relationships could give insight into the encoding of cellular goal states and might enable the leveraging of these dependencies for targeted therapeutic programming.

### Two‐component model of adaptive transcription

**Fig. 3:**
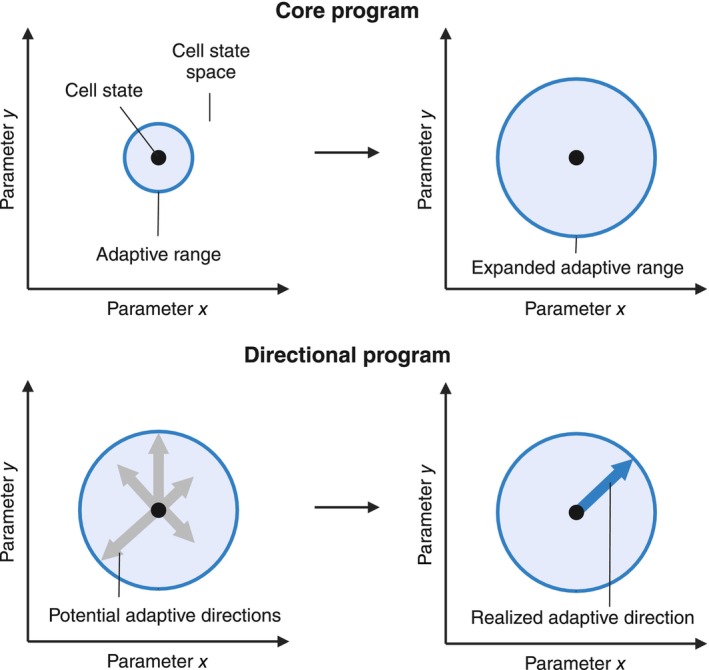
Adaptive transcription consists of a core program and a directional program. Adaptive transcription programs can be divided into a core program (e.g., cAMP response element‐binding protein (CREB) activation and Fos induction) that increases the adaptive range of cells and a directional program (e.g., Npas4 induction) that determines the direction of cellular remodeling. The core program leads to broad cellular changes (e.g., chromatin opening and metabolic flexibility) that increase cellular plasticity and resilience, whereas the directional program induces more concrete changes according to cellular input and in line with higher‐order functions (e.g., upscaling excitatory input onto inhibitory neurons to maintain neural circuit excitation/inhibition balance). The coordinate system represents cellular state space with variables *x* and *y* representing cellular parameters (e.g., expression of a certain protein or a biophysical property such as resting membrane potential). The black dot represents the cell state and the blue circle represents the adaptive range, that is, which states the cell can reach through remodeling. The core program increases the cell's adaptive range (i.e., the radius of blue circle). Out of all possible adaptive directions within this circle, the directional program contributes to the shaping of the adaptation path (i.e., the realized adaptive direction) and which cell state the cell aims for in its remodeling.

In general, there seem to be two components to adaptive transcriptional programs (Fig. 3). The first one is a core program (e.g., induction of Fos and Egr1) that mediates broad changes such as increasing genome‐wide chromatin accessibility after neural activity [243] and is comparable to the previously suggested concept of the ‘generic adaptive gene programme’ [260]. Secondly, it involves a directional program that is dependent on stimulation details and cellular context and guides cellular remodeling and cellular activity patterns into concrete directions (e.g., see differential neuronal gene induction in response to different neuronal activity patterns [261] and different Npas4 target gene programs according to cell type [40] and stimulation input [147]). A hypothesis that is advanced here is that these two components have two distinct, although possibly overlapping, roles in adaptive transcription.

The core component increases the *adaptive range* of a cell, meaning it regulates how much the cell can remodel itself regardless of the adaptive direction. Genes in this category include transcriptional regulators to open chromatin and facilitate read‐out of adaptive gene programs (e.g., transcription factors such as AP1 proteins which have been shown to enhance chromatin accessibility [262]) and proteins that help the cell deal with increased metabolic activity (e.g., increased transcription of monocarboxylate transporter 1 (MCT1) mRNA after stimulation in neurons [263], immune cells [264], and myocytes [265]). The directional component determines the *adaptive direction* into which the reprogramming flows and hence sets more concrete goals and constraints of remodeling. Examples in this group include defined cell–cell communication genes (e.g., Npas4 regulating postsynaptic genes for inhibitory synapses in excitatory neurons and for excitatory synapses in inhibitory neurons [40]).

In addition to the directional program, most cells complement the transcriptional changes through acute changes in their physiology (e.g., synaptic remodeling) that act in concert with transcription and cannot be entirely separated from it. An overlapping function in several adaptive gene programs might hence be to increase molecular supply for cellular computation and remodeling tasks without directly influencing their outcomes. In Aplysia neurons for instance, CREB contributes to different types of synaptic plasticity and activity‐dependent modification of synapses is required in addition to CREB activation for long‐lasting specific plasticity [266]. In addition to transcription, cells also dynamically regulate their translation in response to stimulation, thereby adding an additional layer of computational complexity (e.g., activity‐dependent Arc translation in the regulation of synaptic plasticity [267, 268] and induction of neuronal activity‐dependent microRNAs [269, 270]).

It is thus questionable whether mere overexpression or activation of adaptive transcription core components such as CREB will automatically lead to better or desired organism function. Indeed, while CREB overexpression or activation in the brain has been reported to enhance memory [14, 15, 16, 17, 18, 19, 271], it also leads to memory recall interference [272] and epileptic seizures [273]. Thus, it seems that we may require more than just broad adaptive gene overexpression, and if we do overexpress or activate adaptive transcriptional program components, we have to ensure that cell changes are directed in ways that are beneficial to overall organism function (e.g., through implementing social environment changes in psychiatric therapy or including exercise in the treatment regimen for cardiovascular disease). Identifying and mapping the concrete stimulation parameters at several organizational levels that guide cellular adaptation thus represents an important goal in both basic biology and translational medicine.

### The universality of adaptive transcription

As adaptive transcription components such as CREB, SRF, MEF2, AP1, Egr1, Nr4a1, and others are active in all major organ systems throughout the body in both health and disease, they represent important anchoring points for understanding some of the most fundamental biological processes in animals (e.g., structural and functional plasticity at the cellular level and adaptation at the whole organism level). They also oftentimes allow the conceptual bridging of organizational levels in that molecular alterations have defined effects on tissue organization which in turn leads to differences in organism function and behavior. An example is Npas4's dependency on cellular input [147], subsequent cell‐type‐specific remodeling [40, 147], and specific impact on behavior [39, 147]. Npas4, although it is a single gene, can hence implement higher‐order functions such as excitation–inhibition circuit homeostasis [40]. Similarly, Fos helps shape and stabilize place cell ensembles in the hippocampus of mice during spatial learning [35], thus connecting the induction of a single gene to higher‐order neuronal network processes and cognitive function.

With respect to diseases, adaptive transcription components could represent universal targets to correct organism dysfunction. This follows from their function as facilitators of cell state transitions, as in many medical therapies the goal is exactly to reprogram cells and tissues from a dysfunctional state into a functional one. Therapies that influence adaptive transcription could hence be universal building blocks for correcting organism dysfunction in various therapeutic paradigms. They might be effective supplementary therapies in that they could boost the effects of other targeted therapies. CREB activators for instance could potentiate the effects of antidepressants (because CREB has been shown to mediate antidepressant responses [214]), whereas CREB blockers could support radiotherapies in cancer (because CREB has been demonstrated to mediate radiotherapy resistance [237]). Thus, adaptive transcription activators or repressors could ‘open up’ therapeutic reprogramming windows during which other therapeutics that are more tailored to pathomechanism and target tissue would be potentiated (see below).

### Hyperadaptation in cancer as an instance of an adaptive logic error

Cancer provides an illustrative example of how errors in the logic of transcriptional adaptation discussed above can lead to severe organism dysfunction. Cancer cells can successfully adapt to an enormous amount of internal and external challenges [274] and can hence be considered hyperadaptive. They are metabolically flexible, can evade various treatment efforts and the immune system, and metastasize into tissues that are different from their origin tissue. As we have seen in a previous section, cancer cells use very powerful adaptive transcriptional program components to accomplish these feats. Since cancer cells have a highly unstable genome (e.g., karyotypic aberrations), they must most likely also adapt to internal disturbances and rewire their internal molecular networks to cope with genomic change‐induced transcriptomic and proteomic disorder and it is plausible that adaptive transcription is involved in this process as well.

Interestingly, cells in various cancers express NMDA receptors and the blockade of these receptors reduces several aspects of malignancy [275, 276, 277, 278, 279]. Since neuronal synaptic NMDA receptor activation increases CREB phosphorylation [280] and regulates gene programs that enhance cellular survival [281], it is possible that cancer cells use NMDA receptor‐associated pathways to increase their own resilience and adaptability. In addition, cancer cells and the cells they originate from are also able to undergo transformations in cellular identity, such as in epithelial–mesenchymal transition [282], that is, the transformation of epithelial cells into mesenchymal cell states and CREB has been implicated in this process [283]. SRF can destabilize cellular identity through the suppression of cell‐type specific gene programs [284], and in early postnatal neurons, activity‐regulated enhancer activation mediates a switch in transcription that persists into adulthood [285]. Perhaps one function of adaptive transcription in cancer cells is to facilitate semi‐stable cell state and cell identity transformations. An interesting observation in this regard is that several adaptive genes have been shown to function as oncogenes as well as tumor suppressors (e.g., Egr1 [286] and Atf3 [287]). Thus, in line with their functions in inducing cell state changes, adaptive transcription could perhaps also enable the reprogramming of cancer cells into less malignant phenotypes if the boundary conditions are chosen correctly (see below).

Note that in most instances in oncogenesis, adaptive transcription is fulfilling its immediate physiological role at the cellular level correctly. It helps protect cells against stressors and death and increases their structural and functional flexibility. Thus, there might exist in cancer cells mechanisms that contribute to malignancy in the form of adaptive transcription that are not caused by genomic mutations, but simply by mechanisms that are active in all cells under physiological conditions.

## Leveraging the adaptation code to maintain health and cure disease

A central problem for successfully preventing and treating disease now is this: How can we ensure that cells have the ‘correct’ goal states (i.e., those that render the complete organism as healthy as possible)? We know of several ways to modify cells in the human body such as learning a new skill (e.g., adaptation of connections and excitability of neurons in the brain) or strength training (e.g., hypertrophy of skeletal muscle cells). As explored previously, we also know of several interventions that seemingly reprogram cells (and usually the whole organism) into better functioning states via adaptive transcription such as exercise and cognitive stimulation [1]. Thus, we seem to be able, in these limited circumstances, to enhance cellular function without knowing all the required molecular details. One important consideration is hence the organizational level at which we engage with the system. Under physiological conditions, adaptive transcription is engaged by complex stimuli from the environment [1]. These stimuli lead to the engagement of adaptive transcription components in various tissues and body‐wide adaptations. The transcriptional programs are tightly timed within cells and across tissues (e.g., exercise‐dependent release of the Fndc5 gene product irisin from skeletal muscle into the bloodstream upregulates IEGs in the brain [288]). Single‐target, fixed time‐of‐application pharmacological treatments in contrast lack the complexity associated with natural stimuli and might hence be suboptimal in inducing the necessary cellular reprogramming mechanisms. What is worse, the very adaptive processes that we have discussed here can also dynamically reprogram cellular physiology in order to compensate for pharmacological disturbances to the system thus rendering treatment efforts ineffective. Perhaps, we should hence additionally aim to engage the system at several organizational levels and combine interventions at various scales such as adaptive transcription boosting with environmental stimulation.

### Complexity preservation in therapeutic design

The considerations of the previous paragraph bring us to a central hypothetical concept in leveraging adaptive transcription for health, namely *complexity preservation*. The principle of complexity preservation denotes the effort to preserve as much of the complexity of the natural stimuli that cells and organisms normally react to under physiological conditions as possible when designing artificial approaches to activate adaptation. In social settings for instance, visual experience (e.g., an interesting person) is transmitted into the brain where neurons are exposed to various neurotransmitters and hormones and dynamic membrane depolarization patterns. All these inputs are processed at several levels (e.g., the synaptic membrane and the genome) acting in concert to implement a complex molecular adaptation program within one cell. Then, many cells tune their adaptive programs to each other to form coherent circuits. It seems currently not possible to mimic all these events with simple treatment modalities such as pharmacological compounds acting on one or a few targets. Instead, we might require methods that incorporate these dynamic stimulation patterns that cells react to under natural conditions. This treatment approach can still include pharmacological treatments to boost or block adaptive transcription but it should in any case include complex stimulation patterns such as environmental exposure (social scenarios, exercise, nutrition) or, as we shall see below, at a lower organizational level, bioelectrical programming to achieve desired long‐term goals. Thus, direct adaptive transcription boosters or blockers could serve as building blocks in combinatorial approaches.

### Reprogramming dysfunctional neural circuits with natural environmental stimuli or complex artificial stimuli

One promising potential way of treating disorders like depression and addiction this way might be through changes in the environment, especially with regard to social stimuli in combination with adaptive transcription boosters. Studies in animals show that environmental enrichment induces several IEGs in the brain [289, 290] and diminishes addiction‐like behavior [291] and depression‐like behaviors [292]. Since IEG induction is, in many cases, highly sensitive to the concrete stimulation parameters (e.g., Npas4 is induced differently by different stimuli [147]), it seems advantageous to leverage endogenous signaling mechanisms that implement their own logic to remodel the organism. When considering human patients, it seems however that reprogramming of brains via environmental stimuli requires an initial effort or ability to better one's situation (i.e., to change one's environment) which is, in severe cases, not always possible. Under these circumstances, approaches like electroconvulsive therapy (ECT), which seems effective in the treatment of depression [293], could lead to the induction of cellular plasticity to enable initial remodeling of pathological circuits via adaptive transcription. Indeed, induction of electroconvulsive seizures in the brain of animals has been shown to activate CREB signaling [294] and several IEGs including Bdnf [295], as well as induce synaptic plasticity [296, 297, 298] and reverse behavioral deficits [299, 300]. Thus, ECT might be a good way to maintain some complexity (e.g., differential membrane depolarization patterns and postsynaptic receptor activation through synaptic stimulation) while being accessible to most patients. In cases where pharmacological treatment is indicated, environmental reprogramming approaches that activate adaptive transcription could perhaps be incorporated into treatment, such as CREB activators [301] to enhance complex physiological remodeling while potentially preserving the core dynamics of endogenous activity‐dependent transcription. Indeed, certain antidepressants seem to exert their effects through CREB [214]. It might hence be the case that mere activation of transcriptional adaptation mechanisms might not be enough in disorders like addiction and depression but that the brain also needs inputs that direct rewiring into healthy and more functional states (e.g., through social environmental enrichment). In cases where limited modification of distinct tissues is required, tissue‐level stimulation methods such as electromagnetic field application or pharmacological targeting of tissue‐specific coactivators might be employed.

### Reprogramming cancer

A straightforward way to dealing with cancer that is based on adaptive transcriptional mechanisms might be to simply block their activity (e.g., through AP1 inhibitors [302]). There are, however, several problems when pursuing isolated adaptive transcription component manipulations. The first is that cells could compensate for inhibition of isolated components, such as for instance in the case of CREB knockouts in mice that are compensated through CREM upregulation [303]. Secondly, pharmacological treatments will very likely affect cells in other tissues of the body and since adaptive transcription is important throughout the whole body, its manipulation might entail widespread negative side effects (e.g., CREB blockers, such as those previously developed [304], would perhaps interfere with memory formation and immune defense). In contrast to this, previous work has shown that, for instance, systemic CREB inhibition in mice had no effect on several physiological parameters [305].

One potential way of treating cancer might hence be to reprogram cancer cells into less invasive and destructive ones via physiological or close‐to‐physiological adaptive transcription induction, essentially reprogramming the malignant phenotype (i.e., inducing a cell state transition into a state that is conducive to overall organism survival). As we have seen above, adaptive transcription can be seen as an inducible cell state transition mechanism and SRF for instance can destabilize cell identity [284]. How would one achieve such reprogramming? Ideally, treatments would respect the endogenous dynamics and complexity of natural stimuli that human cells are subjected to in organism development and maintenance. An interesting approach in this regard could be the use of bioelectricity [306]. Cell membrane voltage aberrations have been reported in various cancer types [307] (e.g., depolarization in breast cancer cells [308]) and optogenetically mediated membrane voltage alterations can antagonize tumor formation *in vivo* [309]. As membrane voltage changes are tightly coupled to adaptive transcription in many tissues (see above and Ref. [259]), they might represent a valuable tool to direct tumor cell remodeling (e.g., see current translational approaches for treating cancer based on bioelectricity reviewed in [310]). The leveraging of adaptive transcriptional mechanisms in cancer cells through complex stimulation patterns to reprogram them into states that are less harmful to the overall organism might hence be a novel way to treat cancer. Interesting problems concern the nature of suitable stimulation entities (bioelectricity, engineered cells) and their dynamics (how to choose which stimulation patterns to apply and under what circumstances).

## Conclusions

Adaptive transcription is a crucial mechanism to reprogram cells in response to environmental stimulation. While adaptive gene programs mediate many beneficial functions such as learning and memory, immune defense, muscle hypertrophy, and metabolic adaptation under nutritional scarcity, they are also involved in directing the organism into a dysfunctional state in diseases such as addiction, depression, anxiety, cancer, cardiovascular and metabolic disorders, and autoimmunity. Furthermore, they are crucial in mediating treatment effects in various therapeutic modalities and in counteracting treatment efforts. Adaptive transcriptional programs seem to possess a core program that is activated similarly in nearly all cell types and under most circumstances and a directional program that determines the direction and goal state of cellular reprogramming. Understanding how this goal state is encoded and how one can modify, it will be important for advancing our understanding of animal adaptation and for designing new treatment approaches in medicine. In order to leverage adaptive transcription in therapeutic efforts, it might be advantageous to use complexity‐preserving stimulation (i.e., stimulation that is equal in complexity to that which cells normally react to under physiological conditions) in the form of environmental changes or bioelectrical stimulation in conjunction with single‐molecule targeting of adaptive transcription components. This strategy has the advantage of inducing adaptive transcriptional programs and subsequent organism remodeling with their endogenous dynamics and hence might achieve therapeutic benefits even for complex, multifactorial disorders that are difficult to treat with pharmacological approaches that target single pathways. Adaptive transcriptional programs hence have a widespread role in mediating health and disease, and their elucidation will likely enable profound advances in basic biology and medicine.

## Conflict of interest

The author declares no conflict of interest.
